# Rv0954 Is a Member of the Mycobacterial Cell Division Complex

**DOI:** 10.3389/fmicb.2021.626461

**Published:** 2021-04-20

**Authors:** Ruojun Wang, Sabine Ehrt

**Affiliations:** Department of Microbiology and Immunology, Weill Cornell Medical College, New York, NY, United States

**Keywords:** *Mycobacterium tuberculosis*, cell division, divisome, phosphorylation, Tnseq

## Abstract

Proper control of cell division in the intracellular pathogen *Mycobacterium tuberculosis* is central to its growth, survival, pathogenesis, and resistance to antibiotics. Nevertheless, the divisome components and mechanisms by which mycobacteria regulate their cell cycle are not entirely understood. Here we demonstrate that the previously uncharacterized Rv0954 protein localizes to the mid-cell during cell division and interacts with the division-related proteins LamA, PbpA, and PknH. Deletion of *rv0954* did not result in alterations in cell morphology or sensitivity to cell wall-targeting antibiotics but transposon mutagenesis demonstrated genetic interactions with genes related to cell division. This work suggests that Rv0954 participates in cell division and reveals potential components of the mycobacterial divisome for future investigation.

## Introduction

Our knowledge on how one bacterium becomes two has advanced significantly since Y. Hirota, A. Ryter, and F. Jacob first characterized a collection of thermosensitive *Escherichia coli* (*E. coli*) cell division mutants about half a century ago (Hirota et al., 1968). In the pathogenic mycobacterial species, *Mycobacterium tuberculosis* (Mtb) and *Mycobacterium leprae*, cell division control is an integral part of host-pathogen interactions and may contribute to disease outcomes (Kieser and Rubin, 2014). However, there are many remaining questions to answer and new components to discover in mycobacterial cell division.

Cell division in mycobacteria differs from model rod-shaped bacteria, *E. coli* and *Bacillus subtilis*, in several aspects (Kieser and Rubin, 2014; Baranowski et al., 2019). First, mycobacteria have a complex cell wall consisting of peptidoglycan, arabinogalactan, and mycolic acids (Baranowski et al., 2019). Splitting and synthesizing this highly sophisticated cell envelope at the division site poses a unique challenge for mycobacteria. Second, mycobacteria elongate at the poles in contrast to lateral elongation in the model organisms (Meniche et al., 2014; Cameron et al., 2015). As the division site matures into an elongation site, mycobacteria need to ensure both events are coordinated in time and space by tightly regulating the cell division complex (divisome) and the elongation machinery (Baranowski et al., 2019). Third, model bacteria divide precisely at mid-cell, creating two daughter cells of near-identical length; mycobacteria, on the other hand, grow and divide asymmetrically, leading to daughter cells of varying lengths (Aldridge et al., 2012; Santi et al., 2013). This heterogeneity is promoted by LamA and may be advantageous for the mycobacterial population to survive host stresses and antibiotics treatment (Rego et al., 2017). Mycobacteria’s unusual cell division features likely explain why they lack homologs of many divisome components found in model bacteria and instead encode species-specific factors (Kieser and Rubin, 2014; Baranowski et al., 2019).

Genes with related functions are often located in the same operon allowing co-regulation. The Mtb genome contains many such loci; for example, the *rv0014c* – *rv0018c* region encodes PknA (Rv0015c) and PknB (Rv0014c), two serine/threonine protein kinases that regulate cell division, and their cognate phosphatase, PstP (Rv0018c). The same region also contains *pbpA (rv0016c)* and *rodA* (*rv0017c*), which encode peptidoglycan synthases involved in septum formation during cell division (Sureka et al., 2010). Since *rv0954* is in the same operon with *perM*, which encodes a division protein (Goodsmith et al., 2015; Wang et al., 2019), we hypothesized that Rv0954, a previously uncharacterized protein, may function in cell division or cell wall biosynthesis.

Like *perM*, *rv0954* is one of 219 mycobacterial “core” genes conserved among mycobacterial species and without homologs in other bacteria (Marmiesse et al., 2004). Saturating transposon mutagenesis predicted *rv0954* to be non-essential for growth of Mtb in rich medium (DeJesus et al., 2017), and topology prediction indicated that Rv0954 contains four transmembrane helixes (TMHs) flanked by cytoplasmic N- and C- termini (Supplementary Figure 1). The C- terminal half of Rv0954 (aa 155-303) is rich in proline (18%) and glutamine residues (15%), which often form extended and rigid structures (Williamson, 1994) and may allow Rv0954 to protrude into the cytoplasm for some distance. Here, we examined Rv0954’s localization during the cell cycle, identified its physical interaction partners, and discovered candidate genes that may encode proteins with overlapping functions with Rv0954. Our results support a role for Rv0954 in cell division.

## Materials and Methods

### Bacterial Culture Conditions

*Mycobacterium smegmatis* (Msm) mc^2^155 and derivative strains were cultured in Middlebrook 7H9 medium (BD Difco) containing 0.2% glycerol and 0.05% Tween 80 or Middlebrook 7H10 agar (BD Difco) containing 0.5% glycerol. Mtb H37Rv and derivative strains were cultured in Middlebrook 7H9 medium containing 0.2% glycerol, 0.2% dextrose, 0.5% BSA (Roche), 0.05% Tween 80 or tyloxapol, and 0.085% NaCl or Middlebrook 7H10 agar containing 10% OADC supplement (BD) and 0.5% glycerol. Strains bearing antibiotic resistance cassettes were cultured with hygromycin (50 μg/ml), zeocin (25 μg/ml), kanamycin (25 μg/ml) or streptomycin (20 μg/ml). Growth curves in acidic and low-Mg^2+^ conditions were performed as previously described (Goodsmith et al., 2015; Wang et al., 2019).

### Mutant Construction

The Mtb *Δrv0954* mutant was constructed by allelic exchange using a recombineering approach as previously described (Gee et al., 2012). The *Δrv0954* strain was complemented by introducing a copy of *rv0954* expressed from the *hsp60* promoter into the Mtb genome. We generated the *msmeg_5518* single deletion mutant (Δ*msmeg_5518-perM_msm_:perM_mtb_*) by first integrating *perM*_mtb_ to the attL5 site, then deleting the operon containing *msmeg_5518* and *perM*_msm_. The *rv0954* phospho mutants were constructed by integrating a copy of phospho-ablative or phospho-mimetic Rv0954 fused with C-terminal GFP tags expressed from *p38* promoters into the genome.

### High-Resolution Microscopy

Microscopy imaging was performed using the same methods and equipment as described (Botella et al., 2017). The Mtb samples were fixed with 4% paraformaldehyde for 4 h before removal from BSL-3 containment. Single-cell suspensions were prepared by centrifugation at 800 rpm for 10 min. After spreading on a soft agar pad, the bacteria were visualized using a microscope with appropriate filter sets. For the time-lapse microscopy experiment, we place a drop of Msm single-cell culture (5∼10 μl) to a glass-bottom microwell dish (MatTek, 35 mm, 14 mm microwell). 1% low melting point agarose (UltraPure, Invitrogen) prepared in 7H9 broth was carefully added on top of the bacterial drop. Agarose was left at room temperature to allow solidification before microscopy. We captured snapshots every 15 min and analyzed images with ImageJ (Abramoff et al., 2003).

### Co-immunoprecipitation

We harvested 150 ml of log-phase Mtb cultures and incubated whole-cell lysates with 1% DDM for 2 h before overnight incubation with anti-Flag affinity gel (Sigma) at 4°C. Beads were then washed with lysis buffer (50 mM Tris-HCl, 50 mM NaCl, pH 7.4) and eluted by incubation with 100 ng/μl Flag peptide with gentle rotation for 1 h at 4°C.

To confirm physical interactions, we harvested 30 ml Msm cultures for whole-cell protein lysates, which we incubated with 50 μl anti-GFP mAb-magnetic beads (MBL) overnight shaking at 4°C. After incubation, beads were collected using a DynaMag-Magnet 2 rack (Thermo Fisher Scientific) and washed three times with lysis buffer. Proteins bound to beads were eluted by boiling in Laemmli sample buffer for 10 min and analyzed by immunoblotting.

### Antibiotic Susceptibility Assays

Mtb was grown to early log phase and diluted to an OD of 0.005 in regular 7H9 medium containing 0.2% glycerol, 0.2% dextrose, 0.5% BSA, 0.05% Tween 80, and 0.085% NaCl. Bacteria were then exposed to two-fold dilutions of compounds, and all wells contained 1% DMSO. We recorded MIC as the minimum concentration at which growth was inhibited by at least 90%, compared to controls after approximately 14 days.

### Phosphorylation Assay and Site Identification

We incubated 20 ∼ 40 μg whole-cell protein lysates with 10 U of alkaline phosphatase (Fast AP, Thermo Scientific) for 1 h at 37°C. The same reactions without adding phosphatase served as controls. The samples were then boiled in Laemmli sample buffer and resolved on SDS-PAGE for western blotting. To identify Rv0954 phosphorylation sites, we enriched Rv0954-Flag from Mtb whole-cell lysates by pulldown. Eluates were resolved on SDS-PAGE, and gel pieces at the correct molecular weight range were collected for mass spectrometry identification.

### Transposon Library Construction

The Mtb transposon libraries were constructed by himar1 mutagenesis as previously described (Abramoff et al., 2003). Briefly, approximately 5 × 10^10^ CFU of Mtb were incubated with 10^11^ PFU of MycoMarT7 phage for 4 h at 37°C. The cultures were collected, washed with 7H9 medium containing 0.05% Tween 80, and cultured on 7H9 agar (7H9 medium containing 1.5% bacto agar, 10% OADC, 0.5% glycerol, 0.05% Tween 80, and 25 μg/ml kanamycin) for about 20 days at 37°C. Both WT and *Δrv0954* libraries have TA dinucleotide site coverages of around 50% by Illumina sequencing. Sequencing and data analysis were performed as previously described (Xu et al., 2017).

## Results

### Rv0954 Accumulates at the Mid-Cell Before Septum Formation and Persists Until Cell Constriction

**FIGURE 1:**
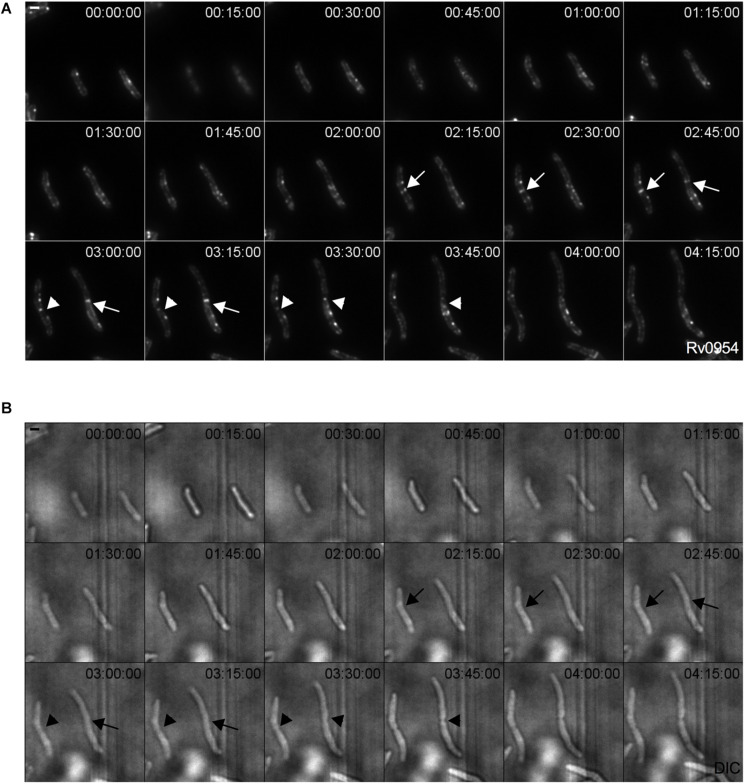
Rv0954 localizes to the mid-cell during cell division. Representative image series from a time-lapse movie of replicating Msm that constitutively express Rv0954-GFP over 4 h and 15 min. Arrows point to mid-cell localization of Rv0954 before cell constriction, and arrowheads point to Rv0954 in cells undergoing constriction. Numbers in the upper right corner indicate time. Scale bar, 1 μm. **(A)** GFP channel. **(B)** Transmitted-light snapshots.

We examined Rv0954’s localization by live-cell imaging using a fluorescent reporter Msm strain. Rv0954 localizes to the membrane in the pre-divisional cells, followed by its accumulation at the mid-cell that lasts until cell constriction (Figure 1). This localization pattern within the cell cycle suggests a role in cell division.

**FIGURE 2:**
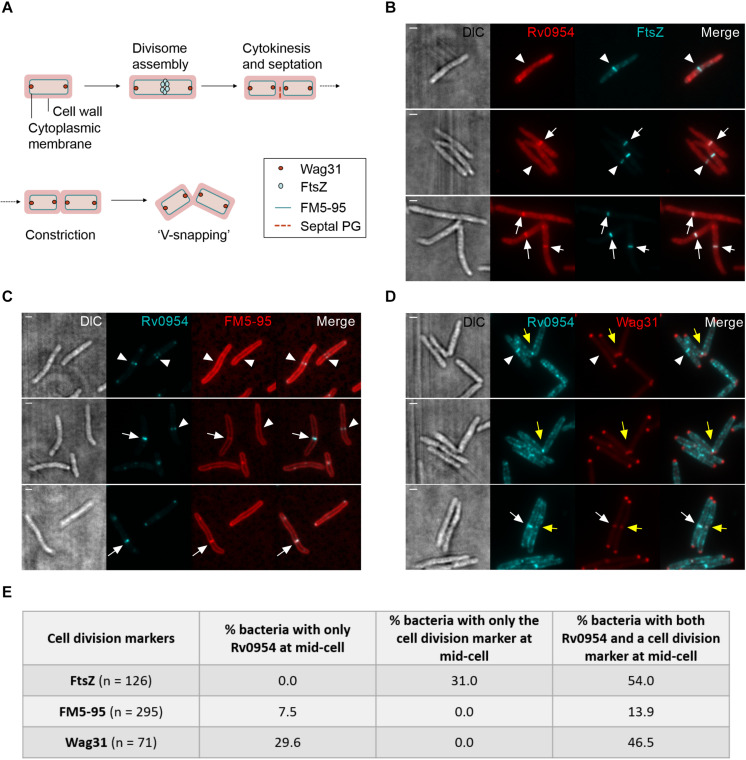
Mid-cell localization of Rv0954 relative to markers of cell division events. **(A)** Schematic of division markers in different stages of cell division. **(B–D)** Representative microscopy images of replicating Msm expressing **(B)** FtsZ_msm_-GFP and Rv0954-mCherry **(C)** Rv0954-GFP **(D)** Rv0954-GFP and Wag31_mtb_-mCherry constitutively. **(B)** Arrowheads point to the presence of FtsZ signals and the absence of Rv0954 signals at the mid-cell. Arrows point to the colocalization of FtsZ and Rv0954. **(C)** Replicating Msm stained with FM5-95 to label the cytoplasmic membrane. Arrowheads point to the presence of Rv0954 signals and the absence of septal FM5-95 staining. Arrows point to the overlapping of Rv0954 and FM5-95 signals at the mid-cell. **(D)** White arrowheads point to the presence of Rv0954 and the absence of Wag31_mtb_ at the mid-cell. White arrows point to the mid-cell localization of both Rv0954 and Wag31_mtb_. Yellow arrows point to Msm undergoing constriction. Scale bar, 1 μm. **(E)** Quantification of bacteria with Rv0954 and respective cell division markers at mid-cell. *N* indicates the number of bacteria quantified.

We next compared the timing of Rv0954’s mid-cell localization to known markers of the cell cycle (Figure 2A). FtsZ is one of the earliest divisome components to assemble at the mid-cell, marking divisome formation (Kieser and Rubin, 2014). Snapshots and quantification of Msm cells containing septal FtsZ bands while lacking mid-cell Rv0954 signals indicates that Rv0954 localizes to the mid-cell after the initiation of divisome assembly (Figures 2B,E). The fluorescent dye FM5-95 stains the cytoplasmic membrane, and its mid-cell signal is indicative of septum formation. In 7.5% cells Rv0954 signals appeared at the mid-cell before septal membranes were visible (Figures 2C,E), indicating that Rv0954’s mid-cell localization precedes septum formation. The mid-cell localization of Wag31 coincides with septum closure, which results in the cytoplasmic compartmentalization of future daughter cells (Santi et al., 2013). Co-localization of Rv0954 and Wag31 at the mid-cell suggested that Rv0954 remains at the septum when cytokinesis is completed (Figures 2D,E). Of particular interest are the cells exhibiting ‘V-snapping’ morphology (Figure 2D, top two panels). Consistent with its role in organizing polar elongation, Wag31 associated with the newly formed poles of future daughter cells (Santi et al., 2013; Kieser and Rubin, 2014). In contrast, Rv0954 localized to the V shape’s inner edge, where the septum is not entirely resolved, perhaps to participate in the final steps of cell separation. Overall, these co-localization experiments demonstrate that the mid-cell localization of Rv0954 precedes septum formation and lasts until cell constriction.

### Rv0954 Interacts With Several Proteins Involved in Cell Division and Cell Wall Biosynthesis

**FIGURE 3:**
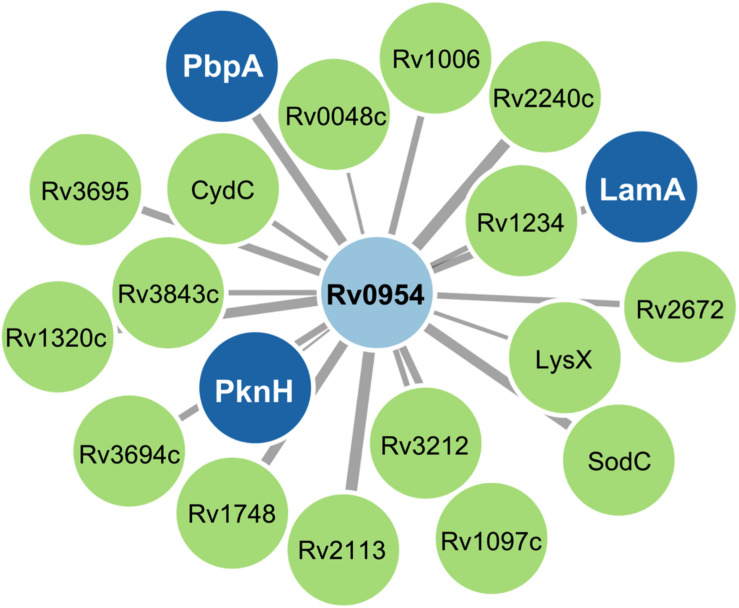
Network map of Rv0954 interacting partners. Cell division-related proteins are marked in blue.

Mycobacterial cell division is mediated by a large protein complex termed ‘the divisome’ (Kieser and Rubin, 2014). To identify protein interactors, we immunoprecipitated Rv0954 from Mtb lysates, followed by mass spectrometry (Figure 3 and Table 1). The 19 identified interactors included a serine/threonine protein kinase involved in cell division regulation: PknH (Sharma et al., 2006; Zheng et al., 2007) and two other known divisome proteins: LamA (also known as MmpS3) and PbpA. LamA is a septal-localizing protein that inhibits cell elongation from the new pole, and PbpA is a transpeptidase that localizes to both the septum and poles (Rego et al., 2017; Viswanathan et al., 2017; Arora et al., 2018). We confirmed the interaction between Rv0954 and PknH in Msm by co-immunoprecipitation (Supplementary Figure 2). The interaction of Rv0954 with these three proteins further supports its involvement in cell division.

**TABLE 1:** Identification of protein interaction partners of Rv0954 in Mtb by mass spectrometry.

Rv#	Gene	Annotation	Sum total spectrum count
Rv1266c*	*pknH*	Serine/threonine-protein kinase H	31
Rv0048c*		Possible membrane protein	20
Rv1640c*	*lysX*	Lysyl-tRNA synthetase	17
Rv3843c*		Probable conserved transmembrane protein	13
Rv1234*		Transmembrane protein	12
Rv3212*		Conserved alanine valine rich protein	12
Rv1620c*	*cydC*	Thiol reductant ABC exporter CydC subunit	12
Rv2672*		Possible secreted protease	11
Rv3694c*		Transmembrane protein	10
Rv1006		Hypothetical protein	10
Rv1097c*		glycine and proline rich membrane protein	9
Rv3695*		Hypothetical protein	9
Rv2198c*	*mmpS3/lamA*	Divisome complex (Rego et al., 2017)	9
Rv1748*		Hypothetical protein	7
Rv0016c*	*pbpA*	Penicillin-binding protein A	7
Rv0432	*sodC*	Periplasmic superoxide dismutase	7
Rv1320c*		Adenylate cyclase	7
Rv2240c*		Hypothetical protein	6
Rv2113*		Integral membrane protein	6

Co-IP of Rv0954 from whole-cell lysates of Mtb *WT:rv0954-flag*. Whole-cell lysates of Mtb containing an empty vector or expressing Rv0060-Flag served as controls. We filtered out the non-specific binding peptides by setting the filter of “Total Spectrum Count” of each replicate to “<4” in the Rv0060-Flag control samples, “<2” in the vector control samples, and “>2” in the Rv0954-Flag samples. The hits were then ranked based on the “Sum Total Spectrum Count” of the Rv0954-Flag samples. The “Sum Total Spectrum Count” is the sum of two independent biological replicates. Annotations of each hit are adapted from PATRIC (https://www.patricbrc.org/) and FLUTE (http://orca2.tamu.edu/U19/). Hits with predicted transmembrane domains are marked with *.

### Deletion of rv0954 in Mtb Did Not Result in Apparent Alterations in Cell Morphology, Growth, or Susceptibility to Cell Wall-Targeting Compounds

**FIGURE 4:**
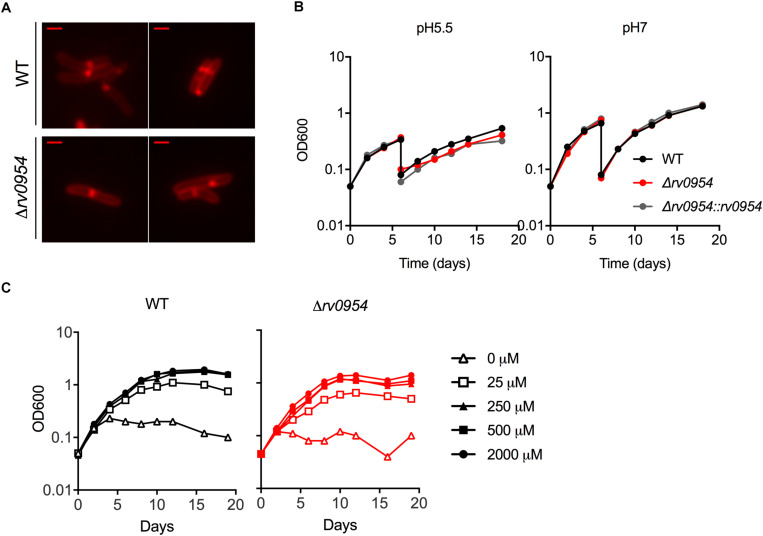
Characterization of Mtb Δ*rv0954*. **(A)** Morphology of replicating WT and Δ*rv0954* cultured in 7H9 broth. The bacterial cells were collected and stained with FM5-95 before imaging. Scale bar, 1 μm. **(B)** Growth curves of Mtb strains sequentially cultured in 7H9 medium adjusted to pH5.5 or pH7. The cultures were diluted in the same medium on day 6. **(C)** Growth curves of Mtb incubated in chelated Sauton’s medium supplemented with Mg^2+^ at the indicated concentrations.

Rv0954 is predicted to be non-essential in Mtb (DeJesus et al., 2017); therefore, we generated a deletion mutant (Supplementary Figure 3). Cells lacking Rv0954 showed no apparent alterations in cell morphology or septum formation (Figure 4A). Deleting *perM*, which codes for a cell division component and is in an operon with *rv0954*, led to growth defects in acidic pH and in reduced Mg^2+^; thus, we tested Δ*rv0954* in the two conditions (Goodsmith et al., 2015; Wang et al., 2019). Acidification slowed Mtb growth, but the Δ*rv0954* mutant showed no growth defect relative to WT Mtb (Figure 4B). The Δ*rv0954* mutant also replicated comparably to WT in medium with reduced Mg^2+^ (Figure 4C). Moreover, Δ*rv0954* tolerated cell envelope targeting compounds similarly to WT (Table 2). These results indicate that the deletion of *rv0954* does not result in significant defects in cell division or cell wall biosynthesis.

**TABLE 2:** Drug susceptibility of H37Rv WT, *Δrv0954*, and complemented strains.

Antibiotic	Target biosynthetic pathway	MIC_90_ (μg/ml)			MIC ratio^a^
		WT	*Δrv0954*	comp	
Ethambutol	Arabinogalactan (Favrot and Ronning, 2012)	1.25	1.25	2.5	1
BTZ043	Arabinogalactan (Favrot and Ronning, 2012)	0.0025	0.0025	0.0025	1
Ethionamide	Mycolic acid (Favrot and Ronning, 2012)	1	0.5	0.5	2
Delamanid	Mycolic acid (Matsumoto et al., 2006)	0.5	0.25	0.5	2
Triclosan	Mycolic acid (Favrot and Ronning, 2012)	37.5	75	37.5	0.5
Isoniazid	Mycolic acid (Favrot and Ronning, 2012)	0.05	0.05	0.05	1
PA-824	Mycolic acid (Favrot and Ronning, 2012)	0.25	0.125	0.25	2
SQ109	Mycolic acid (Sacksteder et al., 2012)	0.625	0.625	0.625	1
Meropenem	Peptidoglycan (Goodsmith et al., 2015)	10	5	10	2
D-cycloserine	Peptidoglycan (Prosser and de Carvalho, 2013)	5	5	5	1
Vancomycin	Peptidoglycan (Goodsmith et al., 2015)	10	5	10	2

*^*a*^MIC for WT/MIC for the *Δrv0954* mutant.*

### Transposon Sequencing (Tnseq) Experiments Revealed rv0954’s Genetic Interactions With Genes Encoding Integral Membrane Proteins

The lack of phenotypes associated with the deletion of *rv0954* in Mtb suggested redundancy in the genome, as is the case for many other cell division components in bacteria, including mycobacteria (Kieser and Rubin, 2014). We expect genes of overlapping functions with *rv0954* to be more critical for Mtb growth and survival when *rv0954* is missing, and therefore transposon mutants of such genes should be underrepresented in the Δ*rv0954* mutant relative to WT Mtb.

**FIGURE 5:**
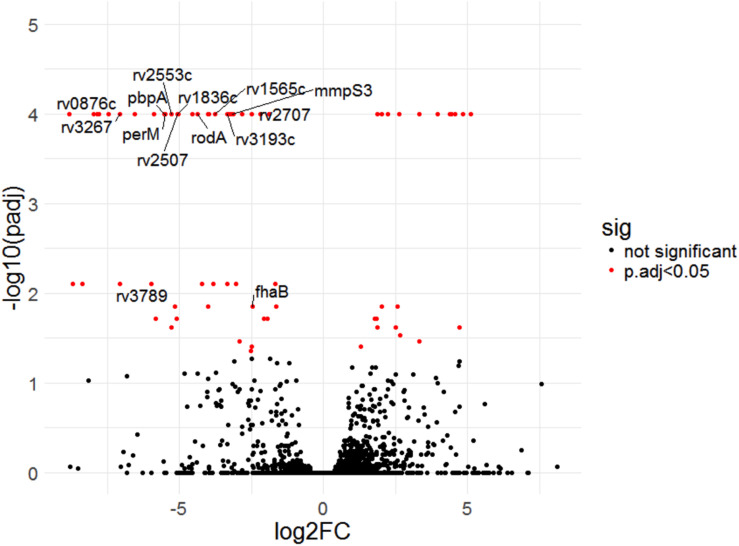
Transposon sequencing (Tnseq) experiments revealed *rv0954*’s genetic interactions with genes encoding integral membrane proteins. Log2 fold change of transposon insertions in WT and the Δ*rv0954* mutant (Δ*rv0954/*WT) plotted against adjusted *p*-values for each genetic locus. Loci with an adjusted *p*-value of <0.05 are colored in red. The dataset was generated from biological duplicates. Annotations of each genetic locus are from publicly available databases PATRIC (https://www.patricbrc.org/) and FLUTE (http://orca2.tamu.edu/U19/). The significantly underrepresented hits encoding integral membrane proteins are indicated by name or gene number.

Four of the top 18 hits underrepresented in the Δ*rv0954* mutant (Table 3) were involved in either cell division or cell wall biosynthesis. PimE (encoded by *rv1159*) is a mannosyltransferase involved in the biosynthesis of phosphatidylinositol mannosides (PIMs), glycolipids in the cell wall of mycobacteria (Morita et al., 2006; Crellin et al., 2008). The deletion of *pimE* in Msm is associated with changes in the cell envelope’s structural integrity and increased sensitivity to toxic compounds, such as certain antibiotics and copper (Morita et al., 2006; Eagen et al., 2018). *Rv0016c* encodes PbpA, a transpeptidase involved in peptidoglycan crosslinking during cell division (Kieser and Rubin, 2014; Viswanathan et al., 2017), and PerM is a divisome component (Goodsmith et al., 2015; Wang et al., 2019). *Rv2553* encodes a putative transglycosylase, and its Msm homolog localized to the septum and poles and interacted with FtsQ (Wu et al., 2018). We further analyzed underrepresented hits that encode integral membrane proteins (Figure 5 and Table 4). Six out of the 14 hits: *pbpA*, *perM*, *rodA*, *rv2553c*, *mmpS3/lamA*, and *fhaB/fipA* encode cell division-related proteins (Sureka et al., 2010; Kieser and Rubin, 2014; Goodsmith et al., 2015; den Blaauwen et al., 2017; Rego et al., 2017; Viswanathan et al., 2017; Arora et al., 2018; Wang et al., 2019). The increased requirements of these genes when Rv0954 is missing supports our hypothesis that Rv0954 is involved in cell division or cell wall biosynthesis.

**TABLE 3:** Underrepresented Tnseq hits in *Δrv0954* as compared with WT.

Rv#	Gene	Annotation
Rv0954		
Rv1252c	*lprE*	Probable lipoprotein
Rv2165c		rRNA subunit methyltransferase H
Rv3215	*entC*	Isochorismate synthase
Rv3593	*lpqF*	Probable lipoprotein
Rv1159*	*pimE*	Mannosyltransferase
Rv0876c		Transmembrane protein
Rv1080c	*greA*	Transcription elongation factor
Rv3267		Hypothetical protein
Rv0238		Transcription regulator
Rv3789		Integral membrane protein
Rv0949	*uvrD1*	ATP-dependent DNA helicase
Rv0016c*	*pbpA*	Penicillin-binding protein A
Rv0384c	*clpB*	Chaperone
Rv0955*	*perM*	Cell division protein (Goodsmith et al., 2015; Wang et al., 2019)
Rv2553c*		Potential cell division protein (Wu et al., 2018)
Rv2507		Proline rich membrane protein
Rv1836c		Hypothetical protein
Rv0211	*pckA*	Phosphoenolpyruvate carboxykinase

Tnseq hits that meet the threshold of adjusted *p*-value <0.01 and log2 fold change <−5. The hits are ranked by log2 fold change from smallest to largest. The dataset is generated from two biological replicates. Annotations of each hit are adapted from PATRIC (https://www.patricbrc.org/) and FLUTE (http://orca2.tamu.edu/U19/) unless otherwise indicated. Genes involved in cell division or cell wall biosynthesis are marked with*.

**TABLE 4:** Underrepresented Tnseq hits in *Δrv0954* that encode integral membrane proteins.

Rv#	Gene	TMH	Annotation
Rv0876c		10	Hypothetical protein
Rv3267		1	Hypothetical protein
Rv3789		4	Integral membrane protein
Rv0016c	*pbpA*	1	Penicillin-binding protein A
Rv0955	*perM*	10	Cell division protein (Goodsmith et al., 2015; Wang et al., 2019)
Rv2553c		1	Potential cell division protein (Wu et al., 2018)
Rv2507		1	Proline rich membrane protein
Rv1836c		1	Hypothetical protein
Rv0017c	*rodA*	12	Cell division protein RodA
Rv1565c		10	Hypothetical protein
Rv3193c		7	Hypothetical protein
Rv2198c	*mmpS3/lamA*	1	Cell division protein (Rego et al., 2017)
Rv2707		6	Hypothetical protein
Rv0019c	*fhaB/fipA*	1	Cell division protein (Sureka et al., 2010; Kieser and Rubin, 2014)

*Tnseq hits that encode proteins with THMs and meet the threshold of adjusted *p*-value <0.05 and log2 fold change <−2. The hits are ranked by log2 fold change from smallest to largest. The dataset is generated from two biological replicates. Annotations of each gene are adapted from PATRIC (https://www.patricbrc.org/) and FLUTE (http://orca2.tamu.edu/U19/).*

### Rv0954 Is Phosphorylated in Mtb

**FIGURE 6:**
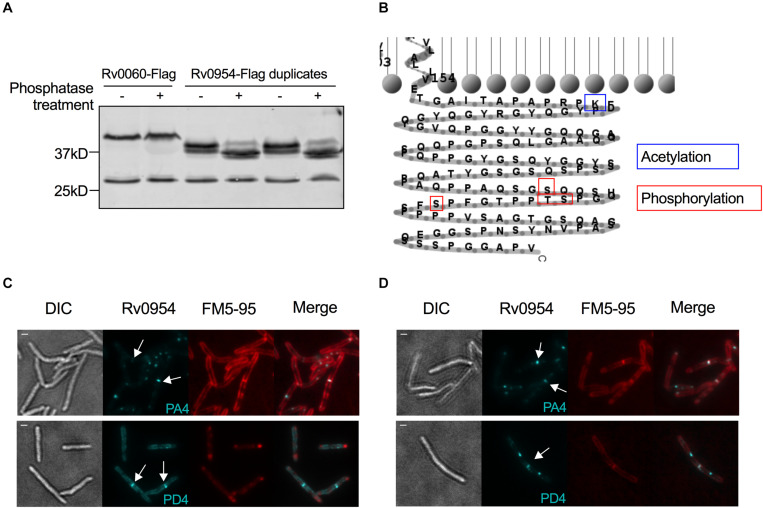
Rv0954 is phosphorylated. **(A)** Detection of Rv0060-Flag (42 kD) and Rv0954-Flag (34 kD) in Mtb whole-cell protein lysates after treatment or not with alkaline phosphatase. The western blot was probed with an anti-Flag antibody. Data are representative of two independent experiments. **(B)** Schematic of the previously reported acetylation (Xie et al., 2015) and newly discovered phosphorylation sites in the C-terminus of Rv0954. **(C,D)** Representative microscopy images of phosphoablative (PA4) or phosphomimetic (PD4) Rv0954 mutants expressed in panel **(C).** WT Msm, or **(D)** Msm Δ*MSEMG_5518-perM_msm_:perM_mtb_*. Msm cells were collected and stained with membrane dye FM5-95 before imaging. Arrows point to the mid-cell localization of Rv0954. Scale bar, 1 μm.

The interaction between Rv0954 and PknH indicated that Rv0954 might undergo phosphorylation (Supplementary Figure 2). To test this hypothesis, we treated protein lysates with phosphatase and examined Rv0954’s migration pattern by western blotting (Figure 6A). Rv0954 migrated as double bands without phosphatase treatment; incubation with alkaline phosphatase down-shifted both bands, indicating that Rv0954 may be subject to phosphorylation and additional modifications, such as acetylation, as previously reported (Xie et al., 2015). The control protein Rv0060 showed no alteration in migration patterns after the same treatment.

We found four phosphorylation sites in the C-terminus of Rv0954 by mass spectrometry: T257, S248, S256, and S264 (Figure 6B). We mutated all four sites to generate phospho-ablative PA4 (Thr/Ser to Ala) or phospho-mimetic PD4 (Thr/Ser to Asp) mutants and evaluated the impact of phosphorylation on Rv0954. Both phospho mutants retained septal localization, either when expressed in addition to the WT MSMEG_5518 homologous allele (Figure 6C) or as the sole source of Rv0954 or MSMEG_5518 (Figure 6D). Our data indicate that Rv0954 phosphorylation does not affect cell division in standard growth conditions yet may play a role in regulating cell division in specific, to be determined physiological conditions.

## Discussion

There are several possible roles that Rv0954 could play during cell division. With four predicted TMHs and a stretched C-terminal domain, Rv0954 could serve as a structural scaffold to recruit additional division proteins. Protein co-localization experiments (Figures 1, 2) showed that Rv0954 lingers at mid-cell from before cytokinesis to cell constriction, possibly recruiting late division proteins such as LamA, which localizes to the mid-cell after FtsZ (Rego et al., 2017), and PbpA. Another possible role of Rv0954 is to anchor intracellular divisome components to the inner cell membrane, similar to the role of ZipA in *E. coli*. In fact, like the proline/glutamine-rich intracellular tail of Rv0954, the cytoplasmic domain of ZipA contains a region that is made of 31% proline and 23% glutamine (Hale and de Boer, 1997). This region may form a long rigid linker to protrude into the cytoplasm for some distance and is proposed to facilitate interaction with FtsZ (Hale and de Boer, 1997). Mycobacteria lack homologs of ZipA, and Rv0954 is unlikely to fill the same role given that FtsZ localizes to the mid cell prior to Rv0954. However, it is plausible that a cytoplasmic division protein other than FtsZ needs membrane attachment via Rv0954.

Rv0954 is phosphorylated in Mtb, possibly by its interaction partner PknH (Figure 3, Table 1, and Supplementary Figure 2). Phosphorylation of division proteins is a major mechanism for mycobacteria to regulate cell division (Kieser and Rubin, 2014; Baranowski et al., 2019). Protein phosphorylation may alter its activity, localization, or interaction with other proteins. The Mtb phospho mutants did not display morphological abnormalities and Rv0954 accumulated at the mid-cell with and without phosphorylation (Figure 6), suggesting that phosphorylation may play an accessory role during normal growth. Alternative explanations include additional phosphorylation sites that exist within Rv0954 or phenotypes masked by proteins with overlapping functions.

It is common to find redundancies within the bacterial divisome (de Boer, 2010; Kieser and Rubin, 2014). *E. coli*, for instance, expresses ZipA and FtsA that have overlapping roles in membrane attachment of FtsZ; the Z-ring could form when either ZipA or FtsA was present, but not when both were inactivated (Pichoff and Lutkenhaus, 2002). Functional redundancy may explain Δ*rv0954*’s lack of phenotypes in cell morphology, drug susceptibility, and growth in acidic pH or low magnesium. These results are in contrast to the phenotypes observed when *perM*, which is part of the same operon, was deleted (Goodsmith et al., 2015; Wang et al., 2019). Although we cannot exclude the possibility that *rv0954* may have an essential role *in vivo*, our current data did not motivate infection experiments. Comparing transposon insertion profiles of the Δ*rv0954* mutant to WT Mtb could help reveal overlapping pathways. We detected a reduced number of insertions in several genes that encode integral membrane proteins (Table 4). These include six related to cell division and eight of unknown functions. Future studies of these hits might reveal additional mycobacterial division factors and unmask Rv0954’s functions.

Two cell division genes, *pbpA* and *lamA*, contained fewer transposon insertions in bacteria lacking Rv0954 than WT. PbpA and LamA also physically interacted with Rv0954 in the Co-IP experiment (Figure 3 and Table 1). PbpA is a transpeptidase that synthesizes the septal peptidoglycan; cells lacking PbpA were hypersusceptible to β-lactams (Arora et al., 2018). PbpA interacted with FhaA and CrgA, both are related to peptidoglycan biosynthesis and cell shape maintenance (Plocinski et al., 2011; Viswanathan et al., 2017). LamA is a highly conserved divisome component across mycobacterial species. It inhibits growth at the new cell poles and creates asymmetry. When treated with cell wall-targeting antibiotics such as rifampicin, cells lacking LamA were killed more uniformly and faster (Rego et al., 2017; Logsdon and Aldridge, 2018). Rv0954 mutant did not show increased sensitivity to cell wall-targeting compounds (Table 2), likely because of the functional overlap among divisome components and the complexity in cell division regulation. Future work on the mechanisms by which Rv0954 interacts with PbpA and LamA could help us understand how mycobacteria integrate growth and cell cycle and prioritize antitubercular targets.

The mycobacterial cell wall is the first barrier that protects bacteria from host stresses and antibiotics. Maintenance of cell wall integrity requires properly regulated cell division. Our genetic and biochemical study of a previously uncharacterized protein Rv0954, including its mid-cell localization, physical interactors, mutant phenotypes, and protein modifications, supported Rv0954 as a new component of the mycobacterial divisome.

## Data Availability Statement

The data presented in the study are deposited in the NCBI repository, accession number PRJNA768285; https://www.ncbi.nlm.nih.gov/bioproject/PRJNA678285.

## Author Contributions

RW designed and performed experiments, analyzed data, and drafted the manuscript. SE supervised the project, acquired funding, and reviewed and edited the manuscript. Both authors contributed to the article and approved the submitted version.

## Conflict of Interest

The authors declare that the research was conducted in the absence of any commercial or financial relationships that could be construed as a potential conflict of interest.
