# Supplementary material for: Rv0954 Is a Member of the Mycobacterial Cell Division Complex
Source: Front Microbiol. 2021 Apr 20;12:626461. doi: 10.3389/fmicb.2021.626461 (PMC8093794; doi:10.3389/fmicb.2021.626461)
Supplement: Supplementary file 1 [file Data_Sheet_1.docx]

Supplementary Material


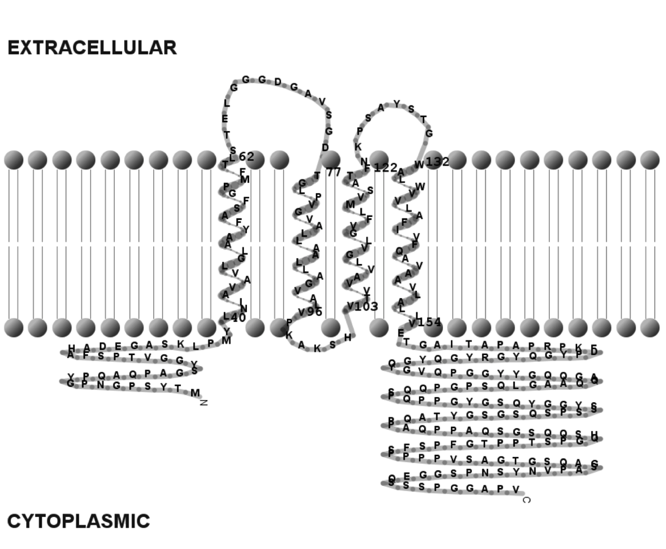


**Supplementary Figure 1. The predicted topology of Rv0954.** TMHMM server (<http://www.cbs.dtu.dk/services/TMHMM/>).


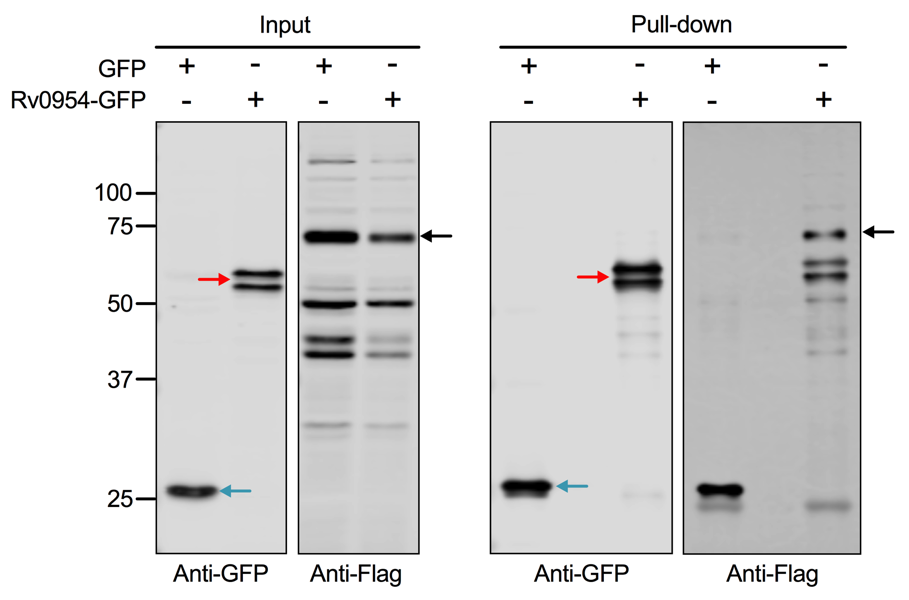


**Supplementary Figure 2. Rv0954 interacts with PknH in vivo.** Co-immunoprecipitation of Rv0954 and PknH_mtb_ from Msm WT*::rv0954-gfp::flag-pknH_mtb_*. Msm WT*::gfp::flag-pknH_mtb_* whole-cell lysates served as a control. We immunoprecipitated GFP-containing proteins and analyzed whole-cell lysates (input) and eluates (pull-down) by western blotting with anti-GFP or anti-Flag antibodies. GFP (cyan arrows), Rv0954-GFP (red arrows), Flag-PknH_mtb_ (black arrows).

**
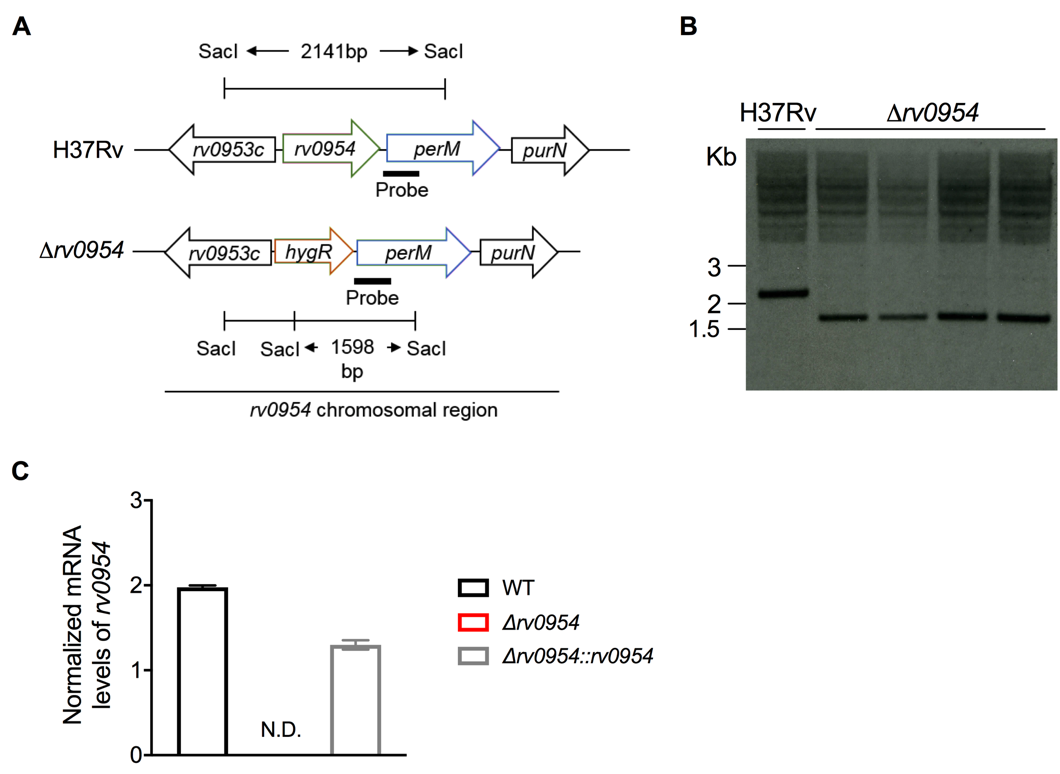
**

**Supplementary Figure 3. Construction of Mtb *Δrv0954*. (A).** Map of the *rv0954* region in WT (H37Rv) and the *Δrv0954* mutant. To construct the knockout mutant, we kept the first 20bp of *rv0954* and replaced the rest with a hygromycin resistance cassette by homologous recombination. **(B).** Southern blots of genomic DNA from WT and four *Δrv0954* candidates after restriction digestion with SacI and hybridization with a probe binds to the region as marked in **(A).** Bands at around 1.5 kb from the four candidates showed the successful deletion of *rv0954.* **(C).** mRNA levels of *rv0954* measured by qRT-PCR*.* RNA was isolated from log phase Mtb cultures. The mRNA levels were normalized to the expression of the housekeeping gene *sigA*. Data are shown as means ± SD of triplicates. N.D. = not detected.

**Supplementary Table 1. Strains used in this work.**

| Species | Strain | Source  or reference | Additional information |
| --- | --- | --- | --- |
| *Mycobacterium tuberculosis* | WT H37Rv | Gift from C. Sassetti, University of Massachusetts |  |
| *Mycobacterium tuberculosis* | $\Delta$*rv0954* | this work | hygromycin resistance cassette replaces *rv0954* |
| *Mycobacterium tuberculosis* | $\Delta$*rv0954::rv0954* | this work | $\Delta$*rv0954 background, hsp60-rv0954 at attL5 site* |
| *Mycobacterium tuberculosis* | WT*::rv0954-flag* | this work | WT background, *p750-rv0954-flag* at attL5 site |
| *Mycobacterium tuberculosis* | WT*+rv0060-flag* | Strain from A. Zaveri (Zaveri et al., 2020) | WT background, *p750-rv0060-flag* expressed from a plasmid |
| *Mycobacterium tuberculosis* | WT*::pTC-MCS* | this work | WT background, empty vector integrated at attL5 site |
| *Mycobacterium smegmatis* | WT*::rv0954-gfp* | this work | WT background, *p38-rv0954-gfp* at attL5 site |
| *Mycobacterium smegmatis* | WT*::rv0954-mCherry+ftsZ-gfp* | this work, pJFR79 plasmid (ftsZ-gfp) from M. Rajagopalan (Dziadek et al., 2003) | WT background, *p38-rv0954-mCherry* at attL5 site, *ftsZ-gfp* expressed from a plasmid |
| *Mycobacterium smegmatis* | WT*::wag31-mCherry::rv0954-gfp* | this work, plasmid containing *wag31-mCherry* was a gift from J. Vaubourgeix, Weill Cornell Medicine | WT background, *p38-wag31-mCherry* at attL5 site*, p38-rv0954-gfp* at tweety site |
| *Mycobacterium smegmatis* | WT*::rv0954-gfp::flag-pknH_mtb_* | this work | WT background, *p38-rv0954-gfp* at tweety site, *hsp60-flag-pknH_mtb_* at attL5 site |
| *Mycobacterium smegmatis* | WT*::gfp::flag-pknH_mtb_* | this work | WT background, *p38-gfp* at tweety site, *hsp60-flag-pknH_mtb_* at attL5 site |
| *Mycobacterium smegmatis* | WT*::rv0954-gfp (PA4)* | this work | WT background, *p38-rv0954-gfp(PA4)* at tweety site |
| *Mycobacterium smegmatis* | WT*::rv0954-gfp (PD4)* | this work | WT background, *p38-rv0954-gfp(PD4)* at tweety site |
| *Mycobacterium smegmatis* | $\Delta$*MSMEG_5518-perM_msm_:: perM_mtb_:: rv0954-gfp(PA4)* | this work | $\Delta$*MSMEG_5518-perM_msm_* background, *p38-perM_mtb_* at attL5 site*, p38-rv0954-gfp(PA4)* at tweety site |
| *Mycobacterium smegmatis* | $\Delta$*MSMEG_5518-perM_msm_:: perM_mtb_:: rv0954-gfp(PD4)* | this work | $\Delta$*MSMEG_5518-perM_msm_* background, *p38-perM_mtb_* at attL5 site*, p38-rv0954-gfp(PD4)* at tweety site |

# Reference

Dziadek, J., Rutherford, S. A., Madiraju, M. V., Atkinson, M. A. L., & Rajagopalan, M. (2003). Conditional expression of Mycobacterium smegmatis ftsZ, an essential cell division gene. *Microbiology*, *149*(6), 1593–1603. https://doi.org/10.1099/mic.0.26023-0

Zaveri, A., Wang, R., Botella, L., Sharma, R., Zhu, L., Wallach, J. B., Song, N., Jansen, R. S., Rhee, K. Y., Ehrt, S., & Schnappinger, D. (2020). Depletion of the DarG antitoxin in Mycobacterium tuberculosis triggers the DNA-damage response and leads to cell death. *Molecular Microbiology*, *114*(4), 641–652. https://doi.org/10.1111/mmi.14571
